# Loquat Leaf Extracts and Obesity-Related Metabolic Dysfunction: Current Evidence, Extraction Technologies, and Combination Strategies

**DOI:** 10.3390/foods15193378

**Published:** 2026-09-23

**Authors:** Chenxiang Sun, Kaiyang Zhu, Yuan Li, Feng Li, Yingxin Zhang, Zhuoyang Wang, Hongyan Zhao, Weiwei Wang, Xianglong Zhang, Wanli Zhang, Qiang Li

**Affiliations:** 1 Food Laboratory of Zhongyuan, Luohe 462300, China; 2 College of Food Science and Engineering, Henan University of Technology, Zhengzhou 450001, China

**Keywords:** *Eriobotrya japonica*, pentacyclic triterpenes, adipogenesis, insulin resistance, subcritical water

## Abstract

Obesity is a multi-factorial metabolic disease for which safe, food-compatible interventions remain of interest. This narrative review evaluates the leaf-specific evidence for *Eriobotrya japonica* in obesity-related metabolic dysfunction and examines its bioactive constituents, extraction technologies, and combination research. Direct studies of loquat leaf preparations were distinguished from indirect evidence obtained with isolated compounds or other plant matrices. Preclinical studies indicate that loquat leaf extracts and triterpenoid-acid fractions can reduce lipid accumulation, body-weight gain, dyslipidemia, insulin resistance, and inflammatory markers in cell and rodent models; however, clinical evidence for obesity is not yet available. Loquat leaves contain triterpenic acids, flavonoids (including flavonols and flavan-3-ols), and phenolic acids, although reported contents vary with origin, harvest stage, and extraction method. Among the technologies reviewed, subcritical water extraction has direct leaf-specific evidence for concentrating corosolic and ursolic acids, whereas evidence for microwave- and enzyme-assisted extraction is less mature or concerns other compound classes. A 1:1 mixture of loquat- and lotus-leaf extracts produced stronger preclinical anti-obesity effects than either extract alone, but formal synergy was not tested; proposed combinations with curcumin or epigallocatechin-3-gallate remain hypotheses. Future work should standardize extracts, link composition to activity, test physical compatibility, and use factorial preclinical designs before clinical translation. Overall, loquat leaf is a promising but still preclinical resource for obesity-related metabolic dysfunction.

## 1. Introduction

Obesity, as a global chronic metabolic disease, has become one of the most serious public health challenges of the 21st century. Data from the World Health Organization show that the global prevalence of obesity has nearly tripled since 1975. Even more concerning is the sharp rise in obesity rates among children and adolescents. A survey indicates that among adolescents, 16.89% are obese, 13.81% are overweight, 43.39% are normal weight, and 25.91% are underweight [1]. This suggests that the disease burden associated with obesity will continue to increase in the coming decades. As shown in Figure 1, obesity is not only an independent disease entity but also an established risk factor for type 2 diabetes [2], metabolic dysfunction-associated steatotic liver disease (MASLD; formerly termed non-alcoholic fatty liver disease) [3,4], hypertension [5], coronary artery disease [6], osteoarthritis [7], and several cancers, including breast and colorectal cancer [8]. Its complex pathophysiological mechanisms involve multiple dimensions, including energy metabolism imbalance, abnormal adipocyte differentiation, chronic low-grade inflammation, insulin resistance, and gut microbiota dysbiosis, making clinical intervention for obesity extremely challenging.

Currently, mainstream strategies for obesity intervention primarily include lifestyle modifications (dietary control and exercise therapy), pharmacotherapy, and bariatric surgery. Although intensive lifestyle intervention is recognized as the first-line foundational therapy, challenges such as poor long-term adherence and high rates of weight regain remain unresolved. While bariatric surgery is highly effective, its applicability is extremely limited due to its invasiveness, high cost, and risk of postoperative complications, confining its use to patients with severe obesity. In the field of pharmacotherapy, although breakthroughs have been made in recent years—such as the introduction of GLP-1 receptor agonists, which offer more significant weight-loss effects than previous options—the associated gastrointestinal adverse effects (nausea, vomiting, diarrhea), potential pancreatic safety risks, and rapid weight regain after discontinuation remain clinical challenges that cannot be overlooked [9]. Representative prescription anti-obesity medications include orlistat and newer incretin-based therapies. Orlistat is generally administered at 120 mg three times daily with fat-containing meals and has been evaluated for obesity management in adolescents aged 12 years and older. Among GLP-1 receptor agonists, semaglutide is initiated at 0.25 mg once weekly and gradually escalated to a maintenance dose of 1.7 or 2.4 mg once weekly; it is approved for long-term weight management in adults and adolescents aged ≥ 12 years with obesity. More recently, the dual GIP/GLP-1 receptor agonist tirzepatide has been introduced for chronic weight management in adults, with a starting dose of 2.5 mg once weekly and maintenance doses of 5–15 mg once weekly. These pharmacotherapies can produce clinically meaningful weight loss, but their indications, age restrictions, tolerability, costs, and need for long-term administration continue to motivate research into safe and food-compatible complementary strategies. Furthermore, centrally acting weight-loss drugs once widely used, such as phentermine and sibutramine, have been partially withdrawn from the market or strictly restricted due to their cardiovascular side effects and neuropsychiatric risks. Therefore, finding a better balance between safety and efficacy and exploring novel anti-obesity agents with mild effects, multi-target regulation, and suitability for long-term intervention have become a shared focus in the fields of nutrition, food science, and pharmacy.

Loquat (*Eriobotrya japonica (Thunb.) Lindl.*) is an evergreen small tree belonging to the genus *Eriobotrya* in the family Rosaceae [10]. Its dried leaves (loquat leaf) constitute a traditional medicinal and edible homologous material in China, included in the Pharmacopoeia of the People’s Republic of China. Characterized by its slightly cold nature and bitter taste, it is attributed to the lung and stomach meridians, with traditional functions including clearing the lung to stop cough and descending adverse qi to alleviate vomiting. Its medicinal use in China spans over two thousand years [11]. Modern pharmacological studies have revealed that loquat leaf is rich in various bioactive components, including triterpenic acids, flavonoids, and polyphenols, exhibiting multiple pharmacological activities such as anti-inflammatory, antioxidant, anti-tumor, hypoglycemic, and lipid metabolism-regulating effects [12]. In recent years, a growing body of research has focused on the potential of loquat leaf extract in weight loss and lipid reduction. It exerts preventive and ameliorative effects on obesity and related metabolic disorders through various pathways, including regulating adipocyte differentiation, promoting lipid oxidation, improving insulin sensitivity, and modulating gut microbiota composition, demonstrating promising application prospects.

Although research on loquat leaf in the field of weight loss and lipid reduction is increasing, the relevant findings are relatively scattered, involving complex and diverse mechanisms of action. Furthermore, numerous challenges remain regarding extraction processes, in vivo bioavailability, and clinical translation. Therefore, this review aims to systematically summarize the weight-loss and lipid-lowering mechanisms of loquat leaf, starting from its main active components (triterpenic acids, flavonoids, polyphenols). These mechanisms include the regulation of lipid metabolism, improvement of insulin sensitivity, modulation of the intestinal microecology, and inhibition of oxidative stress and inflammation. Additionally, this paper analyzes the advantages, disadvantages, and application prospects of key extraction technologies such as subcritical water extraction, enzyme-assisted extraction, ultrasonic-assisted extraction, and microwave-assisted extraction. Finally, it discusses the current challenges and future development directions, aiming to provide a scientific basis and theoretical support for the high-value utilization of loquat leaf resources and the development of novel functional products for weight loss and lipid reduction.

## 2. Literature Search and Evidence Classification

To better interpret and evaluate the applicability and rigor of the literature, relevant studies were searched in the Web of Science Core Collection. This database includes the Science Citation Index Expanded (SCI-Expanded) from 1991 to the present and the Social Sciences Citation Index (SSCI) from 2001 to the present. Boolean operators (AND and OR) and wildcards were used to retrieve the literature containing different combinations of topics, including titles, abstracts, keyword combinations, and author keywords. The search was conducted on 11 March 2026, using combinations of search terms including “*Eriobotrya japonica*” or “loquat”, “leaf” or “leaves”, as well as “obesity”, “lipid metabolism”, “metabolic syndrome”, “triterpene”, “flavonoid”, “phenolic”, “extraction”, and “mixture”. Reference lists of relevant primary studies and reviews were also screened. Leaf-specific chemical, cell, animal, and human studies were prioritized. Studies of isolated compounds from other sources were retained only when the compound had been analytically identified in loquat leaf; these studies are labeled as indirect mechanistic evidence. Studies using other plant matrices for extraction were included only when they addressed the same target compound class and when direct loquat-leaf data were unavailable, and their transferability is discussed as a limitation. Fruit- or seed-only studies were not used as evidence for loquat leaf efficacy. Among the five primary studies that anchor the obesity-focused efficacy, triterpenoid-extraction, and combination discussion, four directly evaluated loquat-leaf material: two preclinical efficacy studies of an extract or purified fraction, one dried-leaf triterpenoid-extraction study, and one loquat-leaf–lotus-leaf mixture study. The fifth efficacy study used an E. japonica cell-suspension preparation and is considered related evidence, not leaf-specific evidence. Other loquat-leaf chemical or extraction-feasibility studies are outside this focused count.

## 3. Leaf-Specific Composition and Evidence for Obesity-Related Outcomes

Loquat leaf contains triterpenoids, flavonoids, phenolic acids, sesquiterpene glycosides, polysaccharides, and other constituents [13,14]. To keep the present review focused on obesity-related outcomes, Table 1 includes compounds only when (1) their occurrence in loquat leaf has been supported by leaf-specific chemical analysis and (2) direct evidence from a loquat leaf preparation or reproducible metabolic evidence for the isolated compound is available. Flavonoids are a subclass of polyphenols; catechins are therefore classified as flavan-3-ols within the flavonoid group, whereas chlorogenic and gallic acids are phenolic acids. Evidence is classified as direct when the tested material was loquat leaf or a defined leaf fraction and as indirect when the experiment used an isolated compound from another source. Indirect evidence can support biological plausibility, but it cannot by itself establish the efficacy of loquat leaf extract. Direct evidence from loquat leaf preparations is very limited. One study showed that a 50% ethanol loquat leaf extract reduced lipid accumulation and adipogenic transcription factors in 3T3-L1 adipocytes and lowered body weight and plasma triglycerides in ob/ob mice [15]. Another study using a purified loquat leaf triterpenoid-acid fraction reduced body-weight gain, visceral adiposity, serum and hepatic lipids, glucose intolerance, oxidative stress, and inflammatory markers in high-fat-diet-fed C57BL/6J mice [16]. In addition, a triterpenoid-rich cell-suspension preparation of *E. japonica* also improved hyperlipidemic and glycemic outcomes in high-fat-fed mice [17], although this material is not equivalent to a conventional leaf extract. Together, these studies support preclinical activity of defined loquat preparations, but differences in extraction, standardization, dose, and model prevent a pooled estimate. No clinical trial has yet established an anti-obesity effect of loquat leaf.

**Table 1:** Representative loquat-leaf constituents, reported amounts, and evidence relevant to obesity-related metabolic dysfunction.

Types	Compound (Formula)	Representative Amount in Loquat Leaf	Obesity-Related Evidence	Evidence Boundary	References
Triterpenic acid	Ursolic acid (C_30_H_48_O_3_)	15.04 ± 0.81 mg/g dry leaf by optimized SWE; 80.70 ± 0.47 mg/g in a purified leaf triterpene-acid fraction	Defined loquat leaf triterpenoid-acid fraction improved adiposity, dyslipidemia, insulin sensitivity, oxidative stress, and inflammation in HFD-fed mice.	Direct for the fraction; isolated-ursolic-acid studies do not establish its independent contribution within the fraction.	[16,18,19]
	Corosolic acid (C_30_H_48_O_4_)	9.63 ± 0.33 mg/g dry leaf by optimized SWE; 129.84 ± 6.26 mg/g in a purified leaf triterpene-acid fraction	Present in the active loquat leaf triterpenoid-acid fraction used in preclinical metabolic studies.	Direct for the multi-acid fraction; compound-specific contribution was not isolated.	[16,18,19]
	Oleanolic acid (C_30_H_48_O_3_)	65.44 ± 1.87 mg/g in a purified leaf triterpene-acid fraction (not raw-leaf content)	The leaf fraction has direct preclinical metabolic evidence; isolated oleanolic acid improved hepatic lipid accumulation in non-loquat studies.	Direct for the fraction; indirect for the isolated-compound mechanism.	[16,19,20]
	Maslinic acid (C_30_H_48_O_4_)	135.24 ± 7.33 mg/g in a purified leaf triterpene-acid fraction (not raw-leaf content)	Isolated maslinic acid activated Sirt1/AMPK and improved steatosis in HFD-fed mice.	Presence is leaf-specific; obesity mechanism is indirect because the compound study did not use loquat-derived material.	[19,21]
Flavonol	Quercetin (C_15_H_10_O_7_)	151.47 ± 71.96 μg/g dry leaf in one methanolic-leaf analysis	Isolated quercetin improved hepatic lipid accumulation and inflammatory markers in HFD-fed mice.	Leaf occurrence is direct; metabolic evidence is indirect and cannot define the effective dose in a leaf extract.	[22,23]
	Rutin (C_27_H_30_O_16_)	558.38 ± 569.83 μg/g dry leaf in one methanolic-leaf analysis	Isolated rutin reduced TNF-α-induced inflammation and altered browning markers in 3T3-L1 adipocytes.	Leaf occurrence is direct; efficacy evidence is indirect and limited to a cell model.	[23,24]
	Kaempferol (C_15_H_10_O_6_)	80.18 ± 49.65 μg/g dry leaf; glycosides were more abundant in the same analysis	Isolated kaempferol improved obesity, inflammation, gut permeability, and microbiota in HFD-fed mice.	Leaf occurrence is direct; metabolic evidence is indirect.	[23,25]
	(+)-Catechin (C_15_H_14_O_6_)	256.19 ± 109.83 μg/g dry leaf in one methanolic-leaf analysis	Tea-catechin mixtures improved metabolic outcomes in HFD-fed mice.	Loquat-leaf catechin occurrence is direct, but tea-catechin composition and exposure are not equivalent.	[23,26]
Phenolic acid	Chlorogenic acid (C_16_H_18_O_9_)	74.40 ± 16.02 μg/g dry leaf in one methanolic-leaf analysis	Isolated chlorogenic acid reduced weight gain and plasma lipids and altered gut microbiota in HFD-fed mice.	Leaf occurrence is direct; metabolic evidence is indirect.	[23,27]

Amounts are representative analytical values, not universal specifications. Values depend on cultivar, origin, harvest stage, drying, solvent, and analytical method. “Purified fraction” values must not be interpreted as raw-leaf content. HFD, high-fat diet; SWE, subcritical water extraction.

To avoid conflating evidence sources, Figure 2 maps the preparation-specific evidence from tested loquat-leaf materials to the corresponding preclinical models and observed metabolic outcomes while presenting compound-informed candidate pathways in a separate tier.

### 3.1. Triterpenic Acids

Triterpenic acids are the primary fat-soluble active components in loquat leaf and are currently recognized as the most promising material basis for its weight-loss and lipid-lowering effects. The triterpenic acids isolated and identified from loquat leaf mainly include ursolic acid, oleanolic acid, and maslinic acid. Among these, ursolic acid and oleanolic acid are the most intensively studied representative components. For instance, Tang et al. [28] investigated the anti-obesity effects of ursolic acid in obese rats induced by a high-fat diet combined with streptozotocin. After 8 weeks of oral administration (2.5, 5, and 10 mg/kg), ursolic acid dose-dependently and significantly reduced blood glucose levels (*p* < 0.001). Compared to the obese control group, ursolic acid decreased plasma insulin and non-esterified fatty acid levels, increased adiponectin levels, and reduced total cholesterol and triglycerides. Mechanistically, ursolic acid reduced oxidative stress in pancreatic tissue by restoring free radical scavenging capacity, inhibited the mRNA expression of *Traf-6*, *Mapk-8*, and *Traf-4*, while enhancing the expression of *Pdx-1*, *Ins-1*, and *Ngn-3*, thereby promoting pancreatic β-cell regeneration and insulin secretion. These results indicated that ursolic acid exerts anti-obesity effects by modulating insulin and JNK signaling pathways. Additionally, Zhang et al. [20] explored the alleviating effects and mechanism of oleanolic acid on high-fat diet-induced lipid accumulation through in vitro and in vivo experiments. In vitro experiments showed that oleanolic acid treatment significantly reduced lipid accumulation in HepG2 cells induced by oleic acid and palmitic acid (confirmed by Oil Red O staining); concurrently, it promoted the nuclear translocation of PPARγ and decreased the expression levels of PPARγ, C/EBP-β, and SREBP-1c. In vivo animal experiment results indicated that dietary intervention with oleanolic acid effectively ameliorated hepatic fat accumulation in mice caused by a high-fat diet and significantly reduced serum levels of the inflammatory cytokines IL-6 and TNF-α. Furthermore, at the experimental concentrations, oleanolic acid caused no pathological lesions in vital organs. Computer simulations further confirmed that oleanolic acid can directly bind to PPARγ, forming a stable conformation. This study provides new experimental evidence for the prevention of non-alcoholic fatty liver disease by oleanolic acid. In addition to ursolic acid and oleanolic acid, maslinic acid has also garnered significant attention in recent years. Liou et al. [21] investigated the ameliorative effects and mechanism of maslinic acid on non-alcoholic fatty liver disease in high-fat diet-induced obese mice. In a 16-week experiment, maslinic acid was administered via intraperitoneal injection for 12 weeks starting from the 5th week. Results showed that compared to the high-fat diet group, maslinic acid significantly reduced body weight, liver weight, and hepatic lipid accumulation in mice, and ameliorated hepatocyte steatosis. Simultaneously, serum concentrations of glucose, leptin, and free fatty acids were significantly decreased in the maslinic acid group, while adiponectin levels were significantly increased. In liver tissue, maslinic acid inhibited transcription factors involved in lipogenic processes and increased the expression of adipose triglyceride lipase. In vitro cell experiments further confirmed that maslinic acid reduces lipid production in hepatocytes by activating the AMPK signaling pathway. This study indicates that maslinic acid combats hepatic steatosis by regulating the activity of enzymes involved in lipogenesis, lipolysis, and fatty acid oxidation in the liver, an effect associated with the modulation of the Sirt1/AMPK signaling pathway. Taken together, triterpenoid acids constitute the best-supported leaf-specific fraction in the current literature. The direct ELTA study [16] links a chemically defined leaf fraction with improvements in adiposity, dyslipidemia, insulin sensitivity, oxidative stress, and inflammation. By contrast, the ursolic-, oleanolic-, and maslinic-acid studies summarized above evaluated isolated compounds and do not demonstrate that the same dose or exposure is achieved after administration of a crude loquat leaf extract. The convergence of AMPK, PPAR-related, and inflammatory pathways is therefore biologically plausible, but interactions among individual triterpenes have not been quantified. Future studies should report the complete triterpene profile, oral exposure, and contribution of individual acids using fractionation or depletion experiments.

### 3.2. Flavonoids

Loquat leaf contains flavonols and their glycosides, including quercetin, rutin, and kaempferol derivatives [14,23]. Their abundance and solubility depend on chemical form and extraction conditions, so they should not be treated as a uniform fraction. Zhao et al. [22] examined isolated quercetin in high-fat-diet-fed obese mice and fatty-acid-loaded HepG2 cells. Quercetin reduced hepatic steatosis and serum TNF-α and IL-6 in mice while lowering cellular lipid accumulation and PCK1 expression in HepG2 cells. Docking suggested binding to PPAR isoforms, although this does not establish target engagement in vivo. Muvhulawa et al. [24] treated TNF-α-challenged 3T3-L1 adipocytes with rutin (10 μM for 6 h). Rutin reduced inflammatory cytokines, limited TNF-α-induced lipolysis, and increased the browning-associated markers PRDM16 and PGC1α; these findings remain confined to a single-cell model. In a 16-week high-fat-diet C57BL/6J mouse model, kaempferol attenuated obesity, fat accumulation, glucose intolerance, and adipose inflammation [25]. Reduced intestinal permeability, immune-cell infiltration, and TLR4/NF-κB signaling accompanied changes in gut microbiota. These compound studies support plausible anti-inflammatory and metabolic mechanisms, but their test materials were not isolated from loquat leaf. A 50% ethanol loquat-leaf extract directly inhibited adipogenesis [15], yet that experiment did not identify the responsible flavonoid or quantify its contribution. Thus, the compound-level findings cannot be transferred to a crude leaf preparation without demonstrating comparable composition and exposure. Fractionation, quantitative profiling, and exposure-matched tests of leaf-derived flavonoids are needed to establish whether they contribute to the observed extract effect.

### 3.3. Phenolic Acids and Flavan-3-ols

Chlorogenic and gallic acids are phenolic acids, whereas catechin and epicatechin are flavan-3-ols within the flavonoid class. Leaf analyses have quantified chlorogenic acid, catechin, and related compounds, with substantial variation by origin and extraction conditions [23]. Wang et al. [27] administered isolated chlorogenic acid (150 mg/kg/day for 6 weeks) to high-fat-diet-fed obese mice and reported lower body weight and plasma lipids, changes in adipogenesis- and lipolysis-related transcripts, and partial reversal of gut microbiota dysbiosis. These results suggest a metabolic contribution from microbiota changes but do not establish that mechanism as causal. In high-fructose-diet diabetic rats, oral gallic acid (30 mg/kg for 4 weeks) lowered fasting glucose, triglycerides, and perirenal fat mass [29]. Changes in adipose insulin-signaling proteins and the lipolytic enzyme ATGL were consistent with improved glucose handling and lipid turnover. Liu et al. [26] studied catechins from green, oolong, and black tea in high-fat-diet-fed mice. Treatment reduced obesity-associated inflammation and hepatic steatosis, increased hepatic PPARα expression, and shifted microbial composition and metabolites, including short-chain fatty acids and lipopolysaccharides. None of these efficacy experiments used a loquat-leaf preparation. Gallic acid is not consistently quantified in leaf-composition studies, and tea catechin profiles cannot be assumed equivalent to those of loquat-leaf flavan-3-ols. The findings therefore provide mechanistic hypotheses rather than evidence that these molecules reach effective exposures through loquat leaf. Testing chemically defined leaf fractions alongside the parent extract would clarify their actual contribution.

## 4. Extraction Technologies: Direct Loquat-Leaf Evidence and Transferability

Extraction determines both the composition and the biological interpretation of a loquat leaf preparation. Conventional water or hydroalcoholic extraction is retained as the benchmark because it is simple, scalable, and used in several direct pharmacological studies [15,16], although it is time-consuming and may be poorly selective. Ultrasound-assisted extraction (UAE), microwave-assisted extraction (MAE), subcritical water extraction (SWE), and enzyme-assisted extraction (EAE) were selected because they represent distinct mass-transfer or cell-wall-disruption mechanisms and have either direct loquat-leaf data or a clear rationale for the compound classes present in the leaf [18,30,31]. Direct loquat-leaf studies are discussed first, whereas studies involving other plant matrices are retained only as process-transfer evidence and cannot be interpreted as proof that identical extraction parameters, yields, or selectivity apply to loquat leaf.

Figure 3 schematically compares the representative equipment configurations and principal physical mechanisms of the extraction technologies discussed in this section. These schemes provide a mechanistic overview and do not imply technological superiority or direct quantitative transferability across plant matrices. Key strengths, limitations, and required next steps for each extraction approach are summarized in Table 2.

### 4.1. Ultrasonic-Assisted Extraction Technology

Ultrasonic-assisted extraction is a technology that utilizes the cavitation effect, mechanical effect, and thermal effect generated by ultrasound in a liquid to disrupt plant cell walls, accelerating solvent penetration and the dissolution of target components. When ultrasound passes through the extraction solvent, alternating compression and rarefaction regions form microscopic bubbles. The growth and instantaneous implosion of these bubbles generate localized high temperature and pressure along with intense shock waves, effectively destroying cell structures and reducing mass transfer resistance [32]. This technology offers advantages such as short extraction time, low operating temperature, reduced solvent consumption, and wide applicability, making it particularly suitable for extracting heat-sensitive components. For instance, Hou et al. [33] used *Chaenomeles speciosa* leaves as raw material, employing ultrasonic-assisted extraction combined with response surface methodology optimization. They determined the optimal extraction process: 93% ethanol, ultrasonic power 390 W, extraction time 30 min, extraction temperature 70 °C, liquid-to-solid ratio 25 mL/g, and two extraction cycles. Under these conditions, the total triterpene yield reached 36.77 ± 0.40 mg/g. After enrichment with X-5 macroporous adsorption resin, the purity of the triterpene fraction increased fourfold to 73.27 ± 0.84%. Thirteen compounds were identified from this fraction using UPLC-QTOF-MS/MS, and five major triterpenes (oleanolic acid, ursolic acid, betulinic acid, maslinic acid, and pomolic acid) were simultaneously quantified using HPLC-QQQ-MS. This study laid the material foundation for the resource utilization and subsequent bioactivity research of *Chaenomeles speciosa* leaves. Xie et al. [34] compared the efficiency of three techniques—ultrasonic-assisted extraction, microwave-assisted extraction, and conventional solvent extraction—for extracting hydroxytyrosol, maslinic acid, and oleanolic acid from olive pomace. The study found that ultrasonic-assisted extraction yielded the highest target compound yields due to its intense cavitation effect and the highest mass and heat transfer efficiency. Through process optimization, the optimal extraction conditions were determined as follows: ethanol concentration 90%, extraction temperature 50 °C, extraction time 5 min, liquid-to-solid ratio 30 mL/g, ultrasonic intensity 135.6 W/cm^2^, and ultrasonic frequency 60 kHz. Greenness assessment showed that ultrasonic-assisted extraction had the lowest E-factor, energy consumption, and carbon emissions, confirming it as the most efficient and environmentally friendly method among the three techniques evaluated. Song et al. [35] used response surface methodology to optimize the ultrasonic-assisted extraction process for total triterpenes from *Ziziphus jujuba* fruits. They established a quadratic regression model involving extraction temperature, ethanol concentration, extraction time, and liquid-to-solid ratio, determining the optimal conditions as follows: extraction temperature 55.14 °C, ethanol concentration 86.57%, extraction time 34.41 min, and liquid-to-solid ratio 39.33 mL/g. Under these conditions, the total triterpene yield reached 19.21 ± 0.25 mg/g. Further analysis indicated that the main triterpenic acids in the jujube fruits were betulinic acid, alphitolic acid, maslinic acid, oleanolic acid, and ursolic acid, and their contents showed a significant positive correlation with antioxidant activity. Bachtler et al. [36] conducted a comparative study on the effectiveness of microwave and ultrasonic-assisted extraction for bioactive compounds from elderberry bark and annatto seeds. For elderberry bark, the optimal process involved using ethanol as the solvent, a liquid-to-solid ratio of 0.01 L/g, and extraction at 55 °C for at least 1 h, under which ultrasonic-assisted extraction yielded approximately 86% of oleanolic acid and ursolic acid. For annatto seeds, the optimal process used ethyl acetate as the solvent, a liquid-to-solid ratio of 0.05 L/g, and extraction for at least 30 min, under which ultrasonication yielded approximately 85% of bixin. Compared to conventional methods, ultrasonic-assisted extraction significantly enhanced the extraction efficiency and yield of the target compounds. Additionally, Amangeldinova et al. [37] employed ultrasonic-assisted extraction (combined with supercritical CO_2_ extraction and subcritical ethanol extraction) to investigate the phytochemical composition of *Rumex confertus* leaves. A total of 53 phytochemical compounds were identified, with gallic acid content reaching as high as 30.71 µg/mg (ultrasonic-assisted extraction-ethanol-4h), rutin content reaching 21.93 µg/mg, and hesperidin content reaching 14.98 µg/mg. The study confirmed the excellent extraction efficacy of ultrasonic-assisted extraction for phenolic acids and flavonoids. Overall, UAE is attractive for loquat leaf because it can shorten extraction at moderate temperature and has direct feasibility data for leaf polysaccharides and phenolic profiling [30,31]. However, the high triterpene yields reported for *Chaenomeles speciosa* or olive pomace cannot be transferred quantitatively to loquat leaf. Solvent composition, acoustic energy density, leaf particle size, and temperature should be optimized together, and the resulting extract should be evaluated for both marker recovery and biological activity. A side-by-side comparison with matched conventional extraction is still required before UAE can be considered superior for obesity-oriented loquat leaf preparations.

### 4.2. Microwave-Assisted Extraction Technology

Microwave-assisted extraction utilizes electromagnetic waves with frequencies between 300 MHz and 300 GHz, enabling polar solvents and polar molecules within plant materials to rapidly absorb microwave energy. This induces dipole rotation and ionic conduction effects, leading to intermolecular friction and heat generation. This volumetric heating method causes the temperature and pressure inside cells to increase rapidly. When the pressure exceeds the tolerance limit of the cell wall, the cells rupture, releasing their contents into the solvent. Compared to traditional heating methods, microwave-assisted extraction offers advantages such as uniform heating, rapid temperature increase, short extraction time, low solvent consumption, and high yield, making it particularly suitable for the rapid extraction of components with good thermal stability [38]. For example, Gómez-Cruz et al. [39] used residual olive pomace from olive processing by-products as raw material to extract high-value triterpenic acids (maslinic acid and oleanolic acid) using microwave-assisted extraction technology. The extraction process using ethanol as solvent was optimized via response surface methodology. The optimal conditions were determined as follows: using unmilled raw material and a solid-to-liquid ratio of 10% (*w*/*v*). Under these conditions, the yields of maslinic acid and oleanolic acid reached 5.1 g/100 g and 2.2 g/100 g of dry basis, respectively. Bachtler et al. [36] compared the effectiveness of microwave-assisted extraction and ultrasonic-assisted extraction for bioactive compounds from elderberry bark and annatto seeds. The results showed that for elderberry bark (using ethanol as solvent), the yields of oleanolic acid and ursolic acid from microwave-assisted extraction were approximately 92%, higher than the 86% achieved by ultrasonic-assisted extraction. For annatto seeds (using ethyl acetate as solvent, liquid-to-solid ratio 0.05 L/g, extraction time 30 min), the yield of bixin from microwave-assisted extraction reached 95%, significantly higher than the 85% achieved by ultrasonication. This study confirmed that microwave-assisted extraction was the optimal auxiliary extraction technology among the three, exhibiting higher extraction efficiency than both conventional methods and ultrasonic-assisted extraction. Boukettaya et al. [40] employed multi-stage microwave-assisted extraction technology to extract total phenols from Tunisian walnut bark and systematically optimized the extraction process. The optimal conditions were determined as follows: microwave power 300 W, extraction repeated 6 times (10 min each), and liquid-to-solid ratio 10 mL/g. Under these conditions, the total phenol recovery rate reached 86%, significantly higher than conventional extraction without microwave irradiation (10 times × 30 min, recovery rate 79%). UHPLC-MS analysis revealed that the main phenolics in the extract were gallic acid, vanillic acid, and quercetin, and the microwave treatment did not alter their qualitative or quantitative composition. MAE can provide rapid volumetric heating and short extraction times, but its performance is highly matrix- and solvent-dependent. The cited olive, elderberry, annatto, and walnut studies demonstrate process capability rather than loquat-leaf efficacy. Direct loquat-leaf reports compiled in the extraction literature suggest that polyphenols, flavonoids, polysaccharides, and oleanolic acid can be recovered by MAE [31], but the available studies do not yet provide a standardized, activity-linked comparison suitable for obesity-oriented products. Local overheating and degradation must be monitored. MAE should therefore be presented as a candidate technology whose optimal conditions require direct confirmation in loquat leaf.

### 4.3. Subcritical Water Extraction Technology

Subcritical water refers to water maintained in a liquid state at temperatures below its critical point (100–374 °C) under sufficient pressure. In this state, the polarity of water decreases significantly as the temperature increases: at room temperature, the dielectric constant of water is approximately 80, indicating strong polarity; when the temperature rises to 200 °C, the dielectric constant drops to about 35, comparable to the polarity level of ethanol at room temperature. This tunable polarity characteristic enables subcritical water to achieve selective extraction of compounds with different polarities, and the entire process uses only water as the solvent, eliminating the need for organic solvents. This makes it an environmentally friendly extraction technology aligned with green chemistry principles [41,42,43,44]. Xiao et al. [45] employed subcritical water extraction technology to extract ursolic acid from *Hedyotis diffusa* and optimized the process parameters using response surface methodology. The optimal extraction conditions were determined as follows: particle size 80 mesh, extraction temperature 157 °C, and liquid-to-solid ratio 30 mL/g. Under these conditions, the maximum yield of ursolic acid reached 6.45 mg/g. Analysis indicated that the influence of each factor on the yield ranked as follows: liquid-to-solid ratio > particle size > temperature. Electrospray ionization time-of-flight mass spectrometry analysis detected a precursor ion peak (*m*/*z* 455.3532) consistent with the theoretical value for ursolic acid in the purified extract, confirming the accurate identification of the target compound. The subcritical water extraction technology established in this study is an efficient and environmentally friendly extraction method. The study also conducted a systematic methodological comparison, analyzing different extraction methods including cold maceration, heat reflux extraction, ultrasonic extraction, microwave-assisted extraction, and subcritical water extraction. The results indicated that subcritical water extraction is a time-saving, economical, and environmentally friendly technique for extracting ursolic acid from *Hedyotis diffusa*. Compared to heat reflux extraction, subcritical water extraction requires shorter time; compared to organic solvent extraction, subcritical water uses water as the solvent, avoiding issues related to organic solvent residues; compared to ultrasonic and microwave-assisted extraction, subcritical water offers better selectivity for extracting less polar triterpenic acids. SWE currently has the clearest direct evidence among the emerging technologies reviewed. Using dried loquat leaves, Mlyuka et al. obtained 25.02 ± 0.71 mg/g total pentacyclic triterpenoids under optimized static-dynamic SWE, compared with 14.39 ± 1.12 mg/g after 4 h conventional extraction; corosolic and ursolic acids reached 9.63 ± 0.33 and 15.04 ± 0.81 mg/g dry leaf, respectively [18]. These results demonstrate selective recovery from the target material rather than extrapolation from another plant. The advantages are reduced organic-solvent use and tunable polarity, whereas the limitations include high temperature and pressure, possible thermal degradation, equipment cost, and scale-up requirements.

### 4.4. Enzyme-Assisted Extraction Technology

Plant cell walls are primarily composed of cellulose, hemicellulose, pectin, and lignin, serving as the main barrier to the dissolution of active components. Enzyme-assisted extraction utilizes enzymes such as cellulase, pectinase, hemicellulase, and β-glucosidase to degrade cell wall structural components or employs specific enzymes to hydrolyze the glycosidic bonds of glycoside compounds, promoting the release of bound components and thereby significantly improving the extraction efficiency of target compounds [46]. This technology features mild conditions, high selectivity, environmental friendliness, and energy efficiency, making it particularly suitable for releasing bound phenolics, flavonoid glycosides, and other components from plant matrices. Wang et al. [47] systematically investigated the application of enzyme-assisted extraction technology in promoting the release of insoluble bound phenolics from guava leaves. Phenolics in food and fruit tree leaves typically exist in three forms: free, soluble conjugated, and insoluble bound. Insoluble bound phenolics are covalently linked to cell wall components, resulting in extremely low bioavailability. The study found that, compared to untreated guava leaves, single xylanase-assisted extraction did not alter the composition or yield of soluble phenolics. In contrast, single cellulase or β-glucosidase-assisted extraction significantly increased the content of soluble phenolics in guava leaves. More importantly, after employing a multi-enzyme complex (cellulase + β-glucosidase + xylanase)-assisted extraction, the soluble phenolic content, flavonoid content, ABTS and DPPH radical scavenging activities, and FRAP ferric reducing antioxidant power were significantly enhanced by 103.2%, 81.6%, 104.4%, 126.5%, and 90.3%, respectively. Interestingly, following the multi-enzyme complex-assisted extraction, phenolics existed predominantly in soluble forms, with almost none remaining in the insoluble bound state, indicating that the multi-enzyme treatment effectively converted bound phenolics into soluble forms. Notably, the contents of quercetin and kaempferol, which possess high biological activity, increased by 3.5-fold and 2.2-fold, respectively. More importantly, the total soluble phenolic extract from guava leaves obtained after multi-enzyme complex-assisted extraction exhibited the highest antioxidant activity and protective effect against supercoiled DNA damage. This study confirms that enzyme-assisted extraction technology can be used to release bound biochemical components from plant matrices, demonstrating broad application prospects in the food and pharmaceutical industries. EAE is most defensible as a mild strategy for disrupting the leaf cell wall and releasing bound polysaccharides or phenolics. Direct loquat-leaf evidence is presently limited and does not yet justify the claim that quercetin, kaempferol, and chlorogenic acid yields will be substantially increased in an obesity-oriented extract. Enzyme identity, activity units, pH, temperature, hydrolysis time, residual enzyme, cost, and batch variation must be reported. Future studies should compare EAE with matched solvent extraction and determine whether increased soluble content translates into improved bioaccessibility and metabolic activity rather than simply a higher colorimetric total.

**Table 2:** Critical comparison of extraction technologies relevant to loquat-leaf bioactives.

Technique	Material	Target/Result	Evidence Level	Main Strength	Main Limitation	Required Next Step	References
Conventional water/hydroalcoholic extraction	Loquat leaf	Direct pharmacology studies used 50% ethanol extracts or defined triterpenoid-acid fractions.	Direct leaf evidence	Simple benchmark; scalable; food-compatible solvent options	Long time, solvent use, limited selectivity; preparation details vary	Standardize marker profile and use as matched comparator for emerging methods	[15,16]
UAE	Loquat leaf; other plant matrices	Direct leaf feasibility for phenolic profiling and polysaccharides; other matrices show recovery of triterpenes/flavonoids.	Direct feasibility plus transfer evidence	Shorter time; moderate temperature; enhanced mass transfer	Acoustic energy is equipment-specific; oxidation/heating possible; other-matrix parameters do not transfer directly	Optimize energy density, solvent, time, and temperature in the same loquat batch; link markers to activity	[33,34,35,36,37]
MAE	Limited direct loquat-leaf reports; other matrices dominate	Potential recovery of polyphenols, flavonoids, polysaccharides, and triterpenes	Limited direct evidence	Rapid volumetric heating; short extraction	Hot spots and degradation; solvent/matrix dependence; scale-up uniformity	Direct loquat-leaf comparison with UAE and conventional extraction under equal input conditions	[38,39,40]
SWE	Dried loquat leaf	25.02 ± 0.71 mg/g total pentacyclic triterpenoids; corosolic acid 9.63 ± 0.33 mg/g and ursolic acid 15.04 ± 0.81 mg/g dry leaf	Direct leaf evidence	Reduced organic solvent; tunable polarity; strong triterpene selectivity	High pressure/temperature, equipment cost, possible thermal degradation	Scale-up, stability, energy analysis, and activity comparison with matched hydroalcoholic extract	[18]
EAE	The loquat-leaf polysaccharide literature; guava-leaf phenolics as transfer evidence	Cell-wall disruption and release of bound material	Limited direct evidence for obesity-relevant loquat constituents	Mild conditions; potential selectivity; low thermal load	Enzyme cost, residual enzyme, pH sensitivity, batch variability; colorimetric totals may overstate useful recovery	Report enzyme units and markers; test bioaccessibility and metabolic activity, not only total content	[46,47]

EAE, enzyme-assisted extraction; MAE, microwave-assisted extraction; SWE, subcritical water extraction; UAE, ultrasound-assisted extraction. Direct evidence means that loquat leaf was the extraction material. Transfer evidence means that another plant matrix was used and the result supports only process plausibility.

## 5. Potential Combination Effects of Loquat Leaf Extract with Other Natural Products

Obesity is a complex disease involving genetic, environmental, behavioral, and metabolic factors. Its pathophysiology includes dysregulation of appetite control, adipose-tissue dysfunction, disturbed hepatic lipid metabolism, gut-microbiota imbalance, and chronic low-grade inflammation. Accordingly, multi-component interventions that act at different points in this network remain of interest. Loquat leaf contains triterpenic acids and flavonoids: the former are associated mainly with AMPK- and PPAR-related lipid metabolism, whereas the latter have been linked to Nrf2- and NF-κB-related oxidative and inflammatory responses. These characteristics provide a rationale for examining loquat leaf in combinations, but shared targets alone do not demonstrate synergy.

Three candidate partners are considered in this section. Lotus leaf was selected because it is another medicinal and food-use leaf material with complementary lipid-metabolism and gut-microbiota research, and a direct loquat-lotus mixture study is available [48]. Curcumin was selected because the cited studies describe well-characterized anti-inflammatory, immunoregulatory, and antioxidant pathways. Green tea extract, particularly epigallocatechin-3-gallate (EGCG), was selected because the cited evidence covers autophagy, pancreatic lipase, insulin signaling, and inflammation. The single-agent studies are presented below as mechanistic context. However, direct combination evidence, indirect mechanistic support, and formulation feasibility are distinguished explicitly.

### 5.1. Combination with Lotus Leaf Extract

Lotus leaf is a traditional medicinal and food-use material whose characteristic constituents include alkaloids such as nuciferine. The pharmacological evidence summarized by Ren et al. [49] indicates that nuciferine acts on several processes relevant to obesity and dyslipidemia. In a high-fat-diet rat model, Dong et al. [50] reported that nuciferine reduced intestinal cholesterol uptake through downregulation of Niemann-Pick C1-like 1, decreased cholesterol esterification, increased hepatic low-density lipoprotein receptor expression, and promoted reverse cholesterol transport and bile-acid excretion through pathways involving PPARα-LXRα-ABCA1 and CYP7A1. These actions differ from, but may complement, the AMPK-related regulation of fatty-acid synthesis and oxidation discussed for the triterpenic acids in loquat leaf. This comparison supports a testable “reduced uptake and synthesis plus increased lipid utilization” model; it does not, by itself, establish an interaction between the two extracts. The anti-inflammatory, antioxidant, and microbiota evidence also remains relevant to the rationale. In the same line of research, lotus-leaf/nuciferine intervention increased antioxidant capacity, altered inflammatory cytokines, and affected serum-extracellular-vesicle signaling [50]. Yu et al. [51] further associated the lipid-lowering action of nuciferine with restoration of gut-microbiota composition, including an increase in *Akkermansia muciniphila*. Loquat-leaf flavonoids and triterpenic acids have been discussed in relation to Nrf2 and NF-κB signaling, while loquat-leaf polysaccharides and polyphenols may influence the intestinal environment. These observations suggest possible complementarity across lipid metabolism, oxidative stress, inflammation, and the gut–liver axis, but most of this evidence comes from separate single-material studies. Importantly, direct preclinical combination evidence is available. Sharma et al. [48] compared a 50% ethanol loquat-leaf extract, a water lotus-leaf extract, and their 1:1 mixture in 3T3-L1 adipocytes and high-fat-diet-fed C57BL/6J mice. The mixture produced larger reductions in lipid accumulation and adipogenic markers in cells and stronger improvements in body-weight gain, tissue weights, hepatic and plasma lipids, and lipogenic protein expression than either individual extract. Thus, the study supports an enhanced combination effect under the tested conditions. It did not include a factorial interaction test, isobologram, combination index, or multiple mixing ratios, so synergy in the strict pharmacological sense cannot yet be concluded. The preparation form should also be considered when translating this result. The two extracts were produced separately and then mixed at a fixed ratio; this is not equivalent to co-extraction or to combining raw-leaf powders. Future studies should standardize marker compounds, compare several ratios with dose-matched single-extract controls, assess chemical and physical stability after mixing, and use factorial analysis to determine whether the observed effect is additive or synergistic.

### 5.2. Combination with Curcumin

Curcumin, the principal polyphenol of turmeric, has documented anti-inflammatory activity [52]. In neuroinflammatory models, curcumin affected TLR4-related signaling and downstream NF-κB responses [53,54], while a review places TLR4 within the broader polyphenol literature [55]. Studies in cigarette-smoke and asthma models also linked curcumin to PPARγ-dependent inhibition of NF-κB [56,57], and reviews discuss its potential to suppress NLRP3 inflammasome activation [58,59]. In a colitis model, curcumin modulated Treg/Th17 balance, a regulatory axis reviewed by Zhang et al. [60,61]. Separate cell studies reported reduced NADPH-oxidase-derived reactive oxygen species and activation of Nrf2-Keap1 antioxidant defenses [62,63]. These findings come from different disease and cell systems, rather than from experiments with loquat leaf. They nevertheless identify endpoints for testing a combination: loquat-leaf triterpenic acids may reduce lipid overload through AMPK-related regulation, whereas curcumin may affect inflammatory and oxidative responses. The overlap in NF-κB, Nrf2, PPAR-related signaling, and inflammatory cytokines offers measurable endpoints, but shared pathway labels alone do not establish a joint effect. Whether these actions are additive or synergistic remains unknown because no direct loquat-leaf–curcumin experiment was identified. Formulation also matters: curcumin’s poor aqueous solubility may cause precipitation or variable exposure when it is mixed with a loquat-leaf extract. Candidate mixtures should be assessed for marker recovery, precipitation, storage stability, and simulated-digestion bioaccessibility before dose-matched single-agent and combination groups are compared using a prespecified interaction analysis.

### 5.3. Combination with Green Tea Extract (EGCG)

The wider green-tea literature covers biological mechanisms, weight control, and insulin resistance [64,65,66]. Epigallocatechin-3-gallate (EGCG) is a major catechin in green tea extract. In diet-induced obese mice, EGCG reduced weight gain, adiposity, hyperlipidemia, and glucose intolerance; in differentiating 3T3-L1 cells, it reduced lipid accumulation and adipogenic markers while suppressing early autophagic flux and changing AMPK-related signaling [67]. By comparison, Zhou et al. [68] observed increased hepatic autophagic flux and lipid-droplet clearance. The differing autophagy findings should be interpreted by tissue and experimental stage. EGCG may also affect intestinal lipid absorption: in simplified intestinal conditions, EGCG-containing aggregates bound and coprecipitated with pancreatic lipase [69], while a mouse study reported 44% lower weight gain and 29.4% higher fecal lipid content together with in vitro lipase inhibition [70]. Other single-agent studies linked EGCG to insulin and inflammatory signaling [71] and miR-143/MAPK7-dependent inhibition of 3T3-L1 cell growth [72]. These results suggest measurable points of possible overlap with loquat-leaf constituents, including AMPK signaling, lipid-droplet clearance, fat digestion, and inflammation. However, none tested EGCG together with loquat leaf, so a combination effect or synergy has not been demonstrated. EGCG is sensitive to pH, oxygen, temperature, light, and metal ions; mixing could alter degradation, precipitation, and bioaccessibility. The proposed formulation should therefore be characterized for EGCG recovery, degradation products, pH, precipitation, and simulated-digestion bioaccessibility before biological testing with dose-matched single-agent groups and a prespecified interaction analysis. Table 3 summarizes the evidence status and validation needs for each proposed combination.

## 6. Challenges and Prospects

Preclinical studies suggest that certain loquat-leaf preparations may improve lipid metabolism, oxidative-stress markers, and inflammation-related outcomes, but the small and heterogeneous evidence base does not establish clinical efficacy. However, despite existing studies revealing the weight-loss and lipid-lowering potential of loquat leaf, this field still faces multi-dimensional systemic challenges: (1) Insufficient depth of basic research, with mechanisms of action not yet systematically elucidated. Current research predominantly focuses on phenotypic observations, lacking in-depth exploration of the synergistic mechanisms among the various classes of active components within loquat leaf extract. How these components interact to regulate multiple metabolic and inflammatory pathways such as AMPK, PPARs, and Nrf2 remains unclear, and research on the structure–activity relationship of the active components is virtually non-existent. (2) Imperfect extraction processes and quality control systems. Although novel extraction technologies are widely applied, existing work primarily concentrates on optimizing single-factor conditions, lacking systematic comparisons between different technologies. Furthermore, research linking extraction processes with biological activity is severely insufficient. Due to the absence of unified quality standards, results from different studies are difficult to compare horizontally or replicate. (3) Combination research is preliminary. A single loquat-leaf–lotus-leaf study reported stronger preclinical effects of a 1:1 mixture than either extract alone [48], but formal synergy was not tested; proposed combinations with curcumin and green tea extract remain untested. (4) Lagging clinical translation research, hindering application development. Current research is primarily confined to in vitro cell experiments and animal models. Data on pharmacokinetic characteristics, long-term safety evaluation, and validation of weight-loss and lipid-lowering efficacy in humans are extremely scarce. To address the aforementioned issues, future research can seek breakthroughs in the following directions: (1) Deepen mechanistic research. Employ network pharmacology and multi-omics technologies to systematically reveal the synergistic mechanisms of its multi-component, multi-target action at a systemic level, and strengthen structure–activity relationship studies to identify key functional groups. (2) Optimize extraction processes. Systematically compare the effects of different novel extraction technologies on the extraction efficiency and functional activity of active components, establish a quality evaluation system based on “component-activity” correlations, and develop unified quality standards for extracts. (3) Advance research on synergistic combinations. Based on mechanistic complementarity, systematically screen for synergistic effects between loquat leaf extract and other natural products. Validate these synergistic effects through in vitro and in vivo experiments and determine the optimal compatibility ratios. (4) Accelerate the clinical translation process. Improve safety evaluation and pharmacokinetic studies and verify the efficacy and safety in overweight/obese populations through rigorous randomized controlled clinical trials. In the future, with the popularization of systems biology technologies, the maturation of green extraction processes, and the deepening of clinical research, particularly by approaching from emerging perspectives such as the regulation of gut microbiota, the “gut-liver axis,” and the “gut-brain axis,” loquat leaf extract may warrant further investigation as a preclinical candidate for obesity-related metabolic dysfunction.

## 7. Conclusions

Loquat leaf contains triterpenic acids, flavonoids, phenolic acids, and other constituents that justify further study in obesity-related metabolic dysfunction. Direct cell and animal studies of leaf extracts and triterpenoid-acid fractions show improvements in adipogenesis, adiposity, lipid profiles, insulin sensitivity, oxidative stress, and inflammation, but the number of studies is small, and clinical efficacy has not been established. Direct extraction evidence is strongest for subcritical water recovery of leaf triterpenoids; other technologies remain promising but require matched comparisons that link composition with activity. A loquat–lotus 1:1 mixture has shown an enhanced preclinical combination effect, whereas strict synergy and proposed combinations with curcumin or EGCG remain unproven. Accordingly, loquat leaf should be regarded as a promising preclinical resource rather than an established weight-loss intervention. Standardized extracts, transparent evidence grading, formulation testing, factorial combination designs, and carefully staged clinical translation are needed to determine whether this potential can be converted into safe and reproducible products.

## Figures and Tables

**Figure 1:**
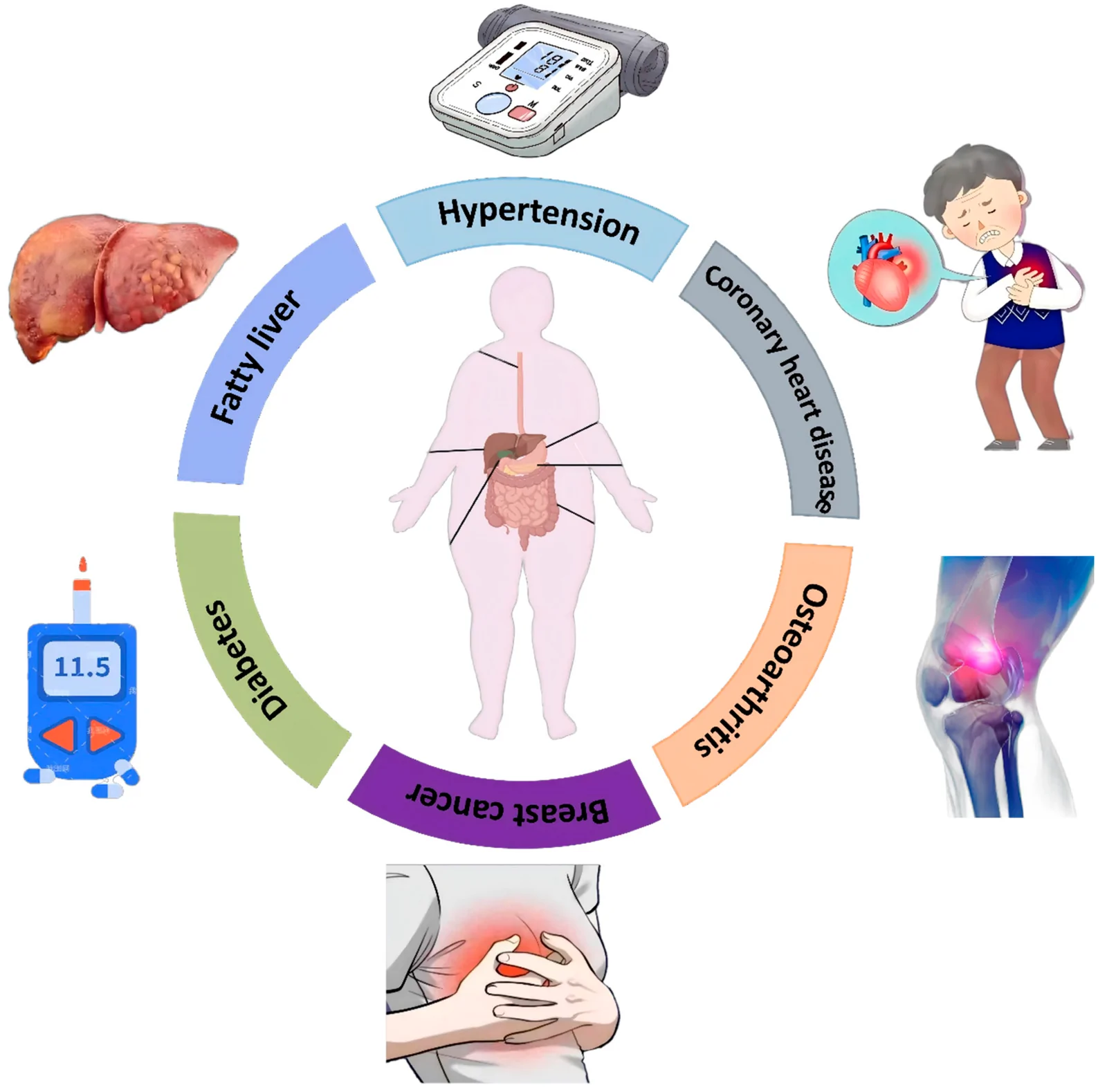
Obesity is a contributing factor to the pathogenesis of various diseases.

**Figure 2:**
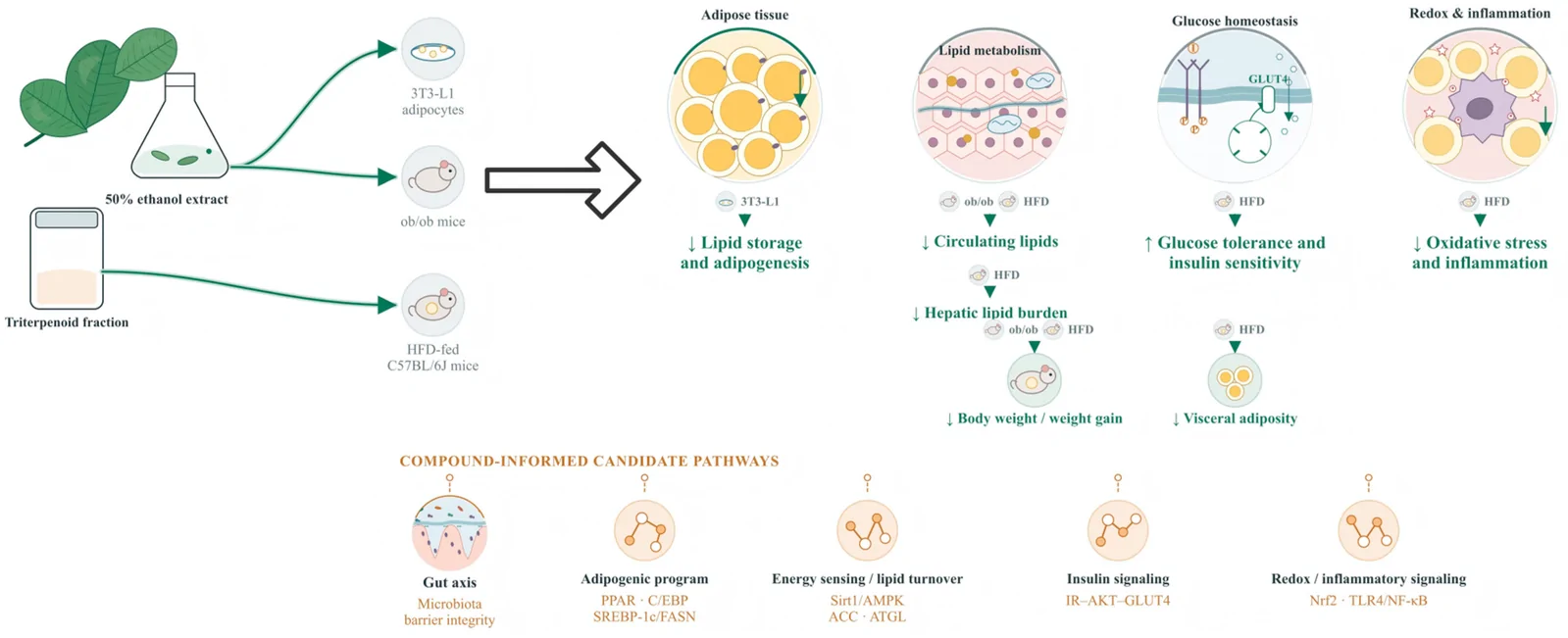
Evidence map linking tested loquat-leaf preparations to preclinical models, observed metabolic outcomes, and compound-informed candidate pathways.

**Figure 3:**
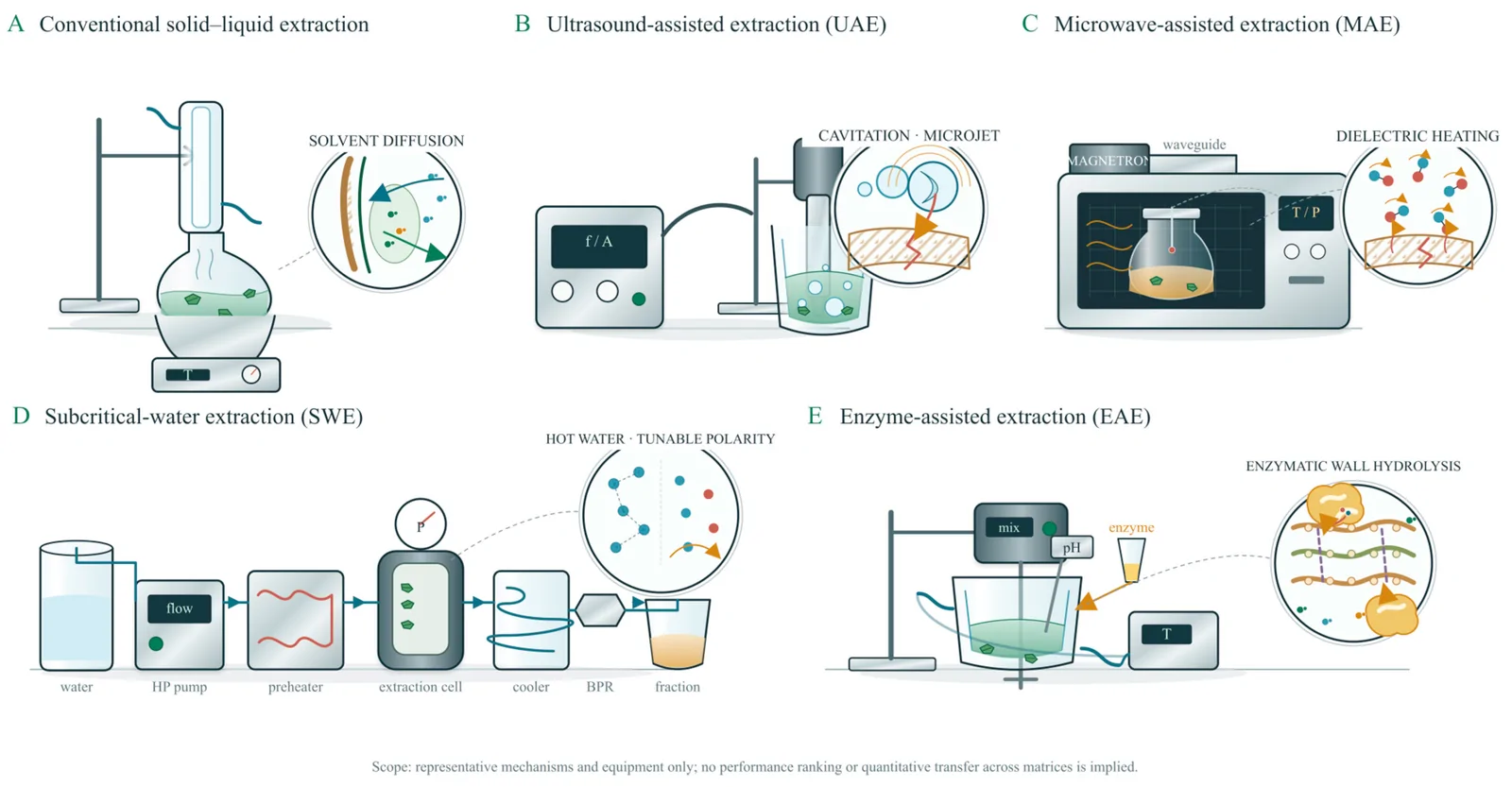
Representative mechanisms and equipment configurations of extraction technologies relevant to loquat-leaf bioactives.

**Table 3:** Evidence status, formulation form, and validation requirements for proposed loquat-leaf combinations.

Candidate	Tested Form/Evidence	Evidence Level	Potential Advantage	Risk/Disadvantage	Validation Required	References
Lotus leaf (*Nelumbo nucifera*)	Separately prepared 50% ethanol loquat-leaf extract and water lotus-leaf extract, mixed 1:1; cell and HFD-mouse comparison with individual arms	Direct preclinical combination evidence; formal synergy not tested	Enhanced effects on lipid accumulation, body-weight gain, tissue lipids, and adipogenic/lipogenic markers versus individual extracts	One fixed ratio; no interaction statistic; marker standardization and product stability incompletely reported	Multiple ratios, dose-matched single arms, factorial interaction analysis, marker recovery, stability, and safety	[48]
Curcumin	No direct loquat–curcumin study identified; mechanistic overlap at AMPK, NF-κB, Nrf2, and inflammation	Hypothesis only	Potential complementary metabolic and anti-inflammatory actions	Poor aqueous solubility; precipitation or phase separation; uncertain oral exposure; formulation may change bioactivity	Solubility, dispersion, precipitation, simulated digestion, pharmacokinetics, then factorial efficacy testing	[53,54,55,56,57,58,59]
Green tea extract/EGCG	No direct loquat–EGCG study identified; mechanistic overlap at AMPK, autophagy, lipase inhibition, and inflammation	Hypothesis only	Potential combined effects on lipid clearance and intestinal fat digestion	EGCG oxidation and pH/temperature/metal sensitivity; color change, precipitation, and dose-dependent tolerability	Chemical stability, particle size, precipitation, bioaccessibility, safety, and dose-matched factorial efficacy testing	[67,68,69,70,71,72]

EGCG, epigallocatechin-3-gallate; HFD, high-fat diet. A stronger mixture effect is not automatically synergy. Synergy requires a prespecified interaction analysis, isobologram, combination index, or comparable quantitative method.

## Data Availability

No new data were created or analyzed in this study. Data sharing is not applicable to this article.

## Abbreviations

The following abbreviations are used in this manuscript:

ACC	acetyl-CoA carboxylase
AMPK	AMP-activated protein kinase
ATGL	adipose triglyceride lipase
C/EBP	CCAAT/enhancer-binding protein
EAE	enzyme-assisted extraction
EGCG	epigallocatechin-3-gallate
GLP-1	glucagon-like peptide-1
HFD	high-fat diet
HPLC	high-performance liquid chromatography
IL	interleukin
MAE	microwave-assisted extraction
MASLD	metabolic dysfunction-associated steatotic liver disease
NF-κB	nuclear factor kappa B
Nrf2	nuclear factor erythroid 2-related factor 2
PPAR	peroxisome proliferator-activated receptor
SCFA	short-chain fatty acid
SREBP-1c	sterol regulatory element-binding protein 1c
SWE	subcritical water extraction
TNF-α	tumor necrosis factor alpha
UAE	ultrasound-assisted extraction
